# Preparation, Characterization of Propargyl Terminal Polybutadiene and Its Crosslinked Elastomers Properties

**DOI:** 10.3390/polym12040748

**Published:** 2020-03-30

**Authors:** Jinxian Zhai, Xiaoyan Guo, Nana Liu

**Affiliations:** School of Materials, Beijing Institute of Technology, Beijing 100081, China; gxy@bit.edu.cn (X.G.); nanaxyh@163.com (N.L.)

**Keywords:** propargyl terminal polybutadiene, polytriazole polybutadiene elastomers, mechanical properties, thermal stability

## Abstract

Propargyl terminal Polybutadiene (PTPB) was successfully prepared through hydroxyl terminal polybutadiene (HTPB) end-capping modification. The FTIR and ^13^C NMR results indicated that the HTPB terminal hydroxyl was thoroughly replaced and yielded the target product, PTPB, with a theoretical propargyl content of 0.66 mmol g^−1^. In comparison with HTPB, PTPB has a lower viscosity. Using 1,6-diazide hexane as a curing agent, polytriazole crosslinked polybutadiene (PT_ri_PB) elastomers with various functional molar ratios (*R*) were prepared by CuAAC reaction, and the glass transition temperatures of the resultant PT_ri_PB elastomers were approximately −75 °C, measured by differential scanning calorimetry (DSC), nearly independent of elastomer *R* values. Mechanical tests indicated, that with the increase in *R*, the mechanical properties of PT_ri_PB elastomers exhibit a parabolic dependence on *R*. In addition, the thermal stability of PT_ri_PB elastomers were also studied. The findings revealed some fundamental features of polytriazole crosslinking elastomer prepared by CuAAC reaction.

## 1. Introduction

Polybutadiene is a very important liquid rubber because of its hydrolytic resistance and higher flexibility at low temperatures, raised from its segment hydrophobic character and low glass transition temperature; it has been used in a wide array of applications, such as for sealants, binders, adhesives, waterproof and anticorrosion coatings, foams, electrical insulation, elastomers, etc. [1]. However, up until now, most crosslinking reactions of polybutadienes still focus on the polyurethane reaction between hydroxyl terminal polybutadiene (HTPB) and isocyanate compounds [2,3]. However, the reaction typically comes with some disadvantages, such as the sensitivity to water and stringent reaction conditions, which easily raise defective network structures and adversely affect their mechanical properties [4].

The cuprous-catalyzed azide-alkyne Huisgen [3+2] dipolar cycloaddition (CuAAC) reaction is the most appealing click chemistry reaction for generating 1,2,3-triazole compounds with the advantages of high efficiency, regioselectivity, mild reaction conditions, no side reaction, insensitive to water, etc., and it has become a versatile synthetic tool [5,6,7,8]. However, because common cuprous compounds are hardly soluble in the organic solvents, and easily lose catalytic efficiency due to their oxidation or disproportion reaction [9], CuAAC reaction is usually carried out under a nitrogen atmosphere, in a solution in order to make cuprous catalyst stable and uniformly dispersed. For instance, Liu dissolved azide-functional polystyrene and 3,5-Bis(propargyloxy)benzyl alcohol in toluene solvent and used the complex of CuBr and PMDETA (n,n,n′,n′′,n′′-pentamethyldiethylenetriamine) as a catalyst. Under a nitrogen atmosphere, the mixture solution was stirred at ambient temperature for 1.5 h, achieving a hydroxyl-containing double-chain polymer [10]. Similarly, Shingu first dissolved CuBr and PMDETA in DMF (dimethylformamide) solvent, and then a solution of triazido-triethynyl-containing polymer in DMF was added into the solution, using a syringe pump, at an extremely slow rate, at 100 °C, under a nitrogen atmosphere. By the intramolecular CuAAC reaction, a µ-ABC tricyclic miktoarm star polymer was prepared [11].

Unlike solution reactions, conventional cuprous catalysts for CuAAC reactions are not fit for the polymer bulk reaction. Moszner mixed multifunctional azides, alkynes and copper (II) acetate together and realized CuAAC bulk step-growth copolymerization in virtue of copper (II) acetate photoreduction under the light irradiation [12]. Schubert directly used CuOAc as the catalyst, and bulk polymerized multifunctional alkynes and azides through CuAAC reaction, and obtained a series of step-growth polymers [13]. However, these polymers contain a great quantity of triazole ring structure with a high glass transition temperature and cannot be used as elastomers. Reshmi used a little amount of cuprous iodide (CuI) solution of acetonitrile as a catalyst and prepared a CuAAC end-crosslinked propargyl terminated polytetramethylene oxide elastomer, using glycidyl azide polymer as a cross-linker [14]. Nevertheless, the strong toxicity of acetonitrile seriously restricts its application scope. 

We envisioned that some cuprous organic complexes could meet the demand of miscibility with prepolymer and proper chemical stability and would be effective catalysts to bulk prepare triazole crosslinked elastomer. Moreover, we found that a copper(I) hexafluoroacetylacetonate cyclooctadiene complex can decrease the activation energy of azide-alkyne cycloaddition from 85 to 46 kJ mol^−1^ and meet conventional requirement of elastomer preparation [15]. Up until now, few studies on bulk CuAAC end-crosslinking polybutadiene elastomers have been reported. In this study, propargyl terminal polybutadiene (PTPB) was synthesized by terminal group modification of HTPB and characterized in detail. Using a cuprous hexafluoroacetylacetonate cyclooctadiene as the catalyst and 1,6-diazide hexane as the curing agent, polytriazole crosslinked polybutadiene elastomers (PT_ri_PB) were prepared through bulk CuAAC reaction, under conventional conditions. Meanwhile, the structures and properties of PT_ri_PB elastomers were also studied in detail.

## 2. Materials and Methods

### 2.1. Materials

The prepolymer used was hydroxyl terminal polybutadiene (HTPB, hydroxyl value 0.68 mmol g^−1^; molecular weight ~6500 g mol^−1^ by gel permeation chromatography) and provided by Liming Research Institute of Chemical Industry. All other agents were purchased and directly utilized without further explication.

### 2.2. Synthesis of 1,6-diazide hexane

Referring to [16], NaN_3_ (1.0 g, 15.0 mmol) was added into a solution of the dichlorohexane (10.0 mmol) in DMF (15.0 mL). The mixture reacted at 60 °C for 10 h, followed by adding distilled water (100.0 mL). The resultant mixture was extracted with ether (3 × 10 mL), and the organic layer was combined and washed three times with distilled water (3 × 10 mL). The solvent ether was removed, and the compound was achieved, whose azide content is 11.9 mmol g^−1^.

### 2.3. Preparation of PTPB

**Scheme 1:**
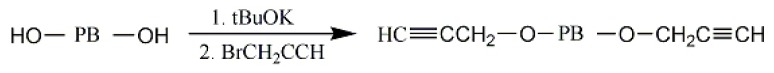
Preparation of PTPB.

Into a three-neck flask fitted with a thermometer, a condenser, a mechanical stirring and nitrogen inlet, 12.5 mmol potassium t-butoxide and 100 mL tetrahydrofuran (THF) were added. After stirring for 30 min at 0 °C, HTPB (14.7 g, 10 mmol hydroxyl) dissolved in 140 mL n-heptane was added and reacted for 3 h. Then, 20 mmol propargyl bromide was added dropwise. After adding propargyl bromide, the resultant mixture was warmed to 30 °C and reacted for another 2 days. The mixture obtained was successively washed twice with 2 × 150 mL brine. The organic layer was separated and evaporated under reduced pressure, to obtain the target product, propargyl terminal polybutadiene (PTPB), at a yield of approximately 14 g. The synthesis route for PTPB is shown in Scheme 1.

### 2.4. Preparation of PT_ri_PB Elastomers

The compositions of polytriazole crosslinked polybutadiene (PT_ri_PB) elastomer are listed in Table 1. All components were mixed uniformly, degassed under vacuum and cured at 50 °C till the characteristic peaks of azide or alkynyl were thoroughly disappeared in virtue of FTIR detection, and PT_ri_PB elastomers crosslinked by bulk CuAAC reaction were obtained. *R* is the mole ratio of the curing agent 1,6-diazide hexane functional group to the PTPB functional group, and the catalyst used was a copper(I) hexafluoroacetylacetonate cyclooctadiene complex. 

### 2.5. Instrumentation

Infrared spectra were recorded on a Nicolet 6700 infrared spectrometer (Waltham, MA, USA). Then, ^13^C-NMR spectra were recorded on a Bruker 600 spectrometer (Switzerland), using gated decoupling, a spectrum width of 20 KHz, a recycle delay of 25 s, a 90° pulse and >500 scans. Chemical shifts were reported in ppm, relative to tetramethylsilane (TMS). The molecular weights of HTPB and PTPB were determined by using a WATERS1515 gel permeation chromatograph (Milford, MA, USA), solution concentration 0.1 mg mL^−1^, column temperature 40 °C, flux 1 mL min^−1^. Viscosity measurements were performed by using a rheostress 300 rheometer (Karlsruhe, Germany) with a 20 mm parallel plate geometry, a gap size 1 mm, a shear rate range 1~100 s^−1^, and collected once every 2 s for 60 s. The glass transition temperatures of PT_ri_PB elastomers were tested by differential scanning calorimetry (DSC, 204 F1, Selb, Germany). All the experiments were carried out in a dry nitrogen atmosphere. The temperature ranged from −110 to 60 °C, with a heating rate of 10 K min^−1^. At room temperature, the mechanical properties of PT_ri_PB elastomers (specification 100 mm × 20 mm × 5 mm) were measured on an Instron 6022 mechanical tester (Boston, MA, USA), at a crosshead speed of 100 mm min^−1^. The thermal gravimetric analysis (TGA) was performed on a Netzsch 209 F1 thermal analyzer (Selb, Germany), under nitrogen atmosphere, at a heating rate of 10 °C min^−1^, from 40 to 800 °C. The samples of 10 mg were used, and the gas flow rate was 60 mL min^−1^.

## 3. Results and Discussion

### 3.1. PTPB Structure

**Figure 1:**
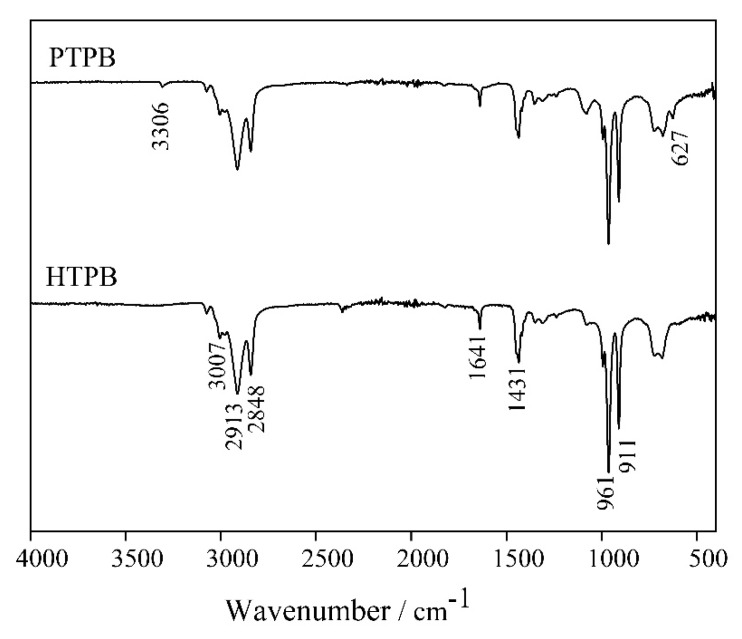
FTIR spectra for HTPB and PTPB.

The FTIR spectra for HTPB and PTPB are shown in Figure 1. HTPB shows major characteristic vibrations, such as C–H stretching vibration at 3007, 2913, and 2848 cm^−1^; C=C stretching vibration at 1641 cm^−1^; and C–H bending vibration at 1431, 961, and 911 cm^−1^. As for O–H stretching vibration peak, it is not easily distinguished because of its lower content within HTPB. These are in good agreement with the report [17]. However, in comparison with HTPB, PTPB presents characteristic peaks of propargyl group at 3306 and 627 cm^−1^ because of the terminal hydroxyl of HTPB having been etherified, in addition to other characteristic peaks of polybutadiene.

**Figure 2:**
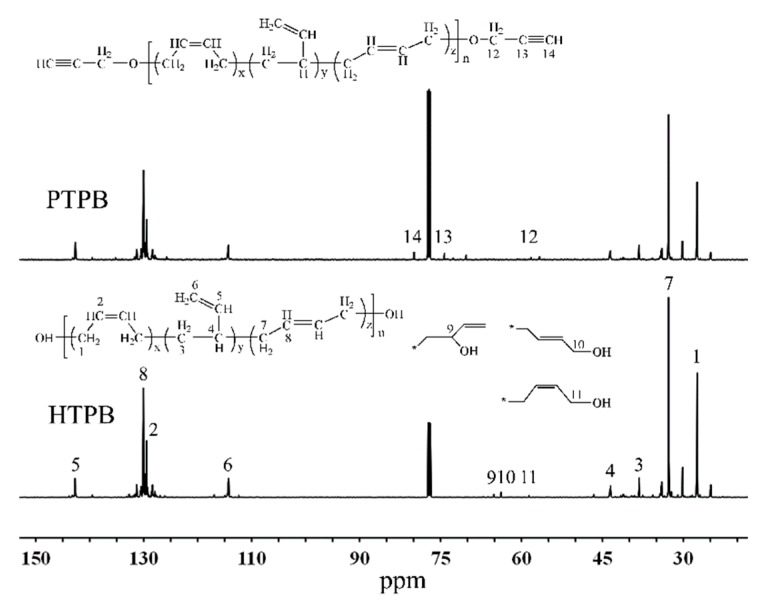
The ^13^C NMR spectra for HTPB and PTPB.

The ^13^C NMR spectra for HTPB and PTPB are shown in Figure 2. Polybutadiene is comprised of the cis/trans-1,4 enchainment, vinyl-1,2 enchainment structures, so it is clear that the peaks at 27.5 and 129.5 ppm are attributed to cis-1,4 enchainment structure, and the peaks at 32.9 and 130.1 ppm are attributed to trans-1,4 enchainment structure. Meanwhile the peaks at 38.3, 43.6, 142.8, and 114.5 ppm are attributed to vinyl-1,2 enchainment structures [18,19]. It should be emphasized that the small peaks at 65.2, 63.8, and 58.6 ppm originate from carbon atoms connected to terminal hydroxyl shown in Figure 2_._

In the ^13^C NMR spectra of PTPB, the typical peaks attributed to polybutadiene backbone still exist at same locations. However, the peaks at 65.2, 63.8, and 58.6 ppm presented in ^13^C NMR spectra of HTPB thoroughly disappear in that of PTPB. Instead, the peaks attributed to the propargyl carbon atom 12, 13, and 14 emerge at 58.3, 74.3, and 79.9 ppm, respectively. These suggest that the terminal hydroxyl of HTPB has been thoroughly etherified, having yielded the target product PTPB.

### 3.2. Propargyl Value

**Figure 3:**
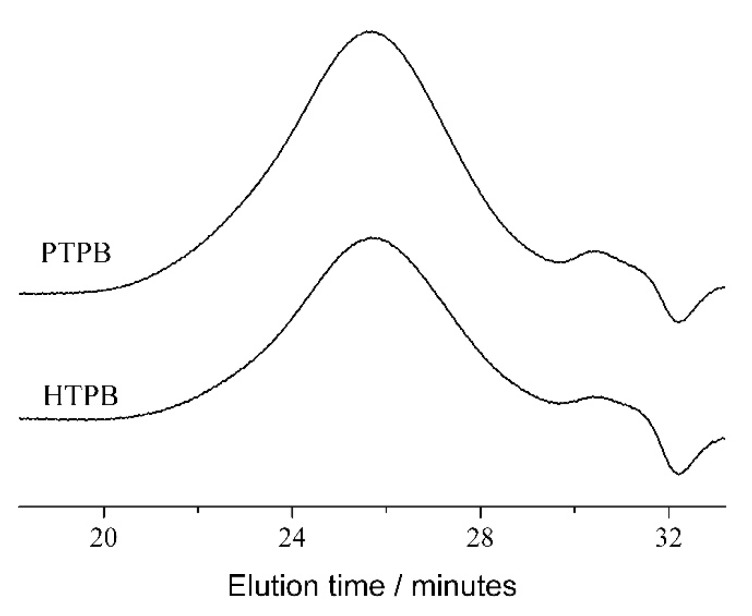
GPC curves for HTPB and PTPB.

Up until now, there has not yet been a directly quantitative analysis method reported for terminal propargyl content. In order to determine the propargyl value of prepolymer PTPB, gel permeation chromatography (GPC) tests were carried out, and the traces of HTPB and PTPB are shown in Figure 3. The two traces are very similar to each other, and the parameters of HTPB and PTPB evaluated by GPC are listed in Table 2. The number average molecular weight of HTPB is 6588 g mol^−1^, and that of PTPB is 6678 g mol^−1^. Moreover, the polydispersity index of HTPB (2.12) is also very close to that of PTPB (2.11). Prepolymer HTPB and PTPB have virtually identical molecular weight and distribution. These suggest that, in the course of PTPB preparation, few chain extension and break reactions occurred, and HTPB was transformed into PTPB only through end-capping modification. In combination with ^13^C-NMR results, it can be inferred that the terminal group content of polybutadiene should stay conservative.

According to the given HTPB hydroxyl value (0.68 mmol g^−1^), the corresponding propargyl content of PTPB is evaluated to be 0.66 mmol g^−1^ (see Equation (1)), which was used to determine the R values of preparing PT_ri_PB elastomer (see Section 2.4).

*C*_propargyl_ = *C*_hydroxyl_ / [1+ *C*_hydroxyl_ × (*M*_propargyl_ −1) × 10^−3^] (1)

where, *C*_propargyl_ is the content of propargyl groups in PTPB, mmol g^−1^, *C*_hydroxyl_ is the content of hydroxyl groups in HTPB, mmol g^−1^, and *M*_propargyl_ is the molecular weight of propargyl groups (39 g mol^−1^).

### 3.3. Viscosity

**Figure 4:**
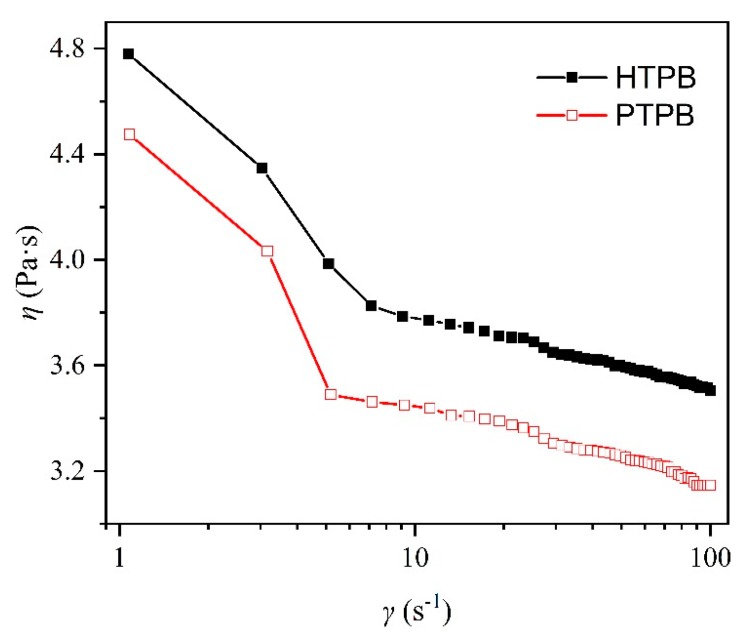
Dependences of viscosity on shear rates for PET and ATPET.

The dependences of viscosities on the shear rates for HTPB and PTPB are shown in Figure 4. The viscosities of both HTPB and PTPB initially rapidly decrease with the increase in shear rates, followed by slow decrease and giving shear-thinning characteristics. Clearly, HTPB has remarkably higher viscosities than PTPB. 

In combination with FTIR, NMR and GPC results, the structure distinguish between HTPB and PTPB only lie in their terminal groups (hydroxyl for HTPB and propargyl for PTPB), so the discrepancy in viscosity of HTPB and PTPB should also come from them. It is well-known that hydroxyls are capable of forming strong hydroxyl bonding interactions with each other, but propargyls are not. This suggests that there still exists hydroxyl bonding within HTPB matrix, having strengthened the interaction among the HTPB chains and exhibiting higher viscosity. In contrast, the terminal propargyl of PTPB has no perceptible interaction with other atoms, and PTPB exhibits a lower viscosity. This is notably beneficial to the process ability of its elastomer preparation.

### 3.4. DSC

**Figure 5:**
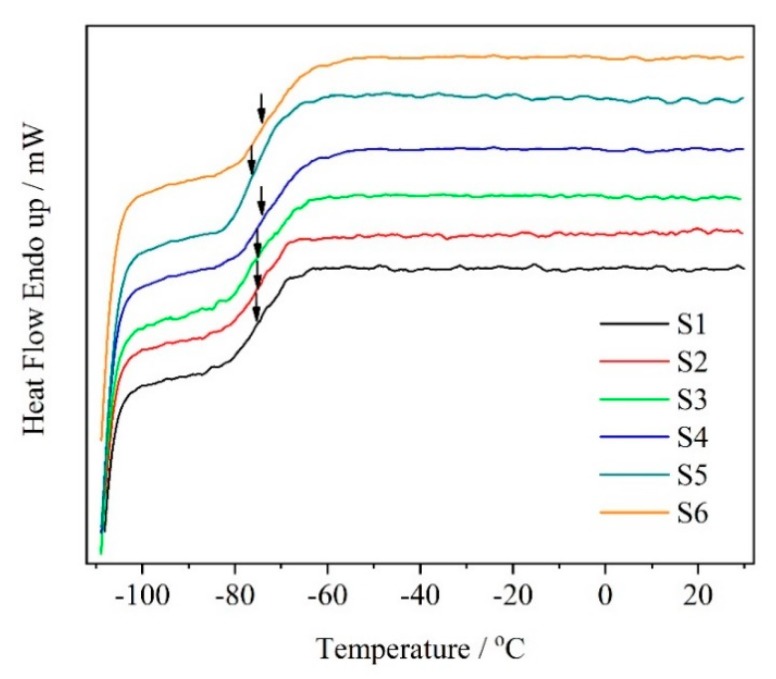
Differential scanning calorimetric (DSC) curves for elastomers.

In virtue of the hydroxyl value (0.68 mmol g^−1^) and the number average molecular weight (6588 g mol^−1^) of HTPB, it can also be inferred that the hydroxyl average functionality of HTPB is about 4; it is greater than 2 as a result of its transfer reactions occurring during preparation process [20]. This implies that the propargyl functionality of PTPB is also about 4, and the multi-functional PTPB can be cured by adopting a di-functional azido compound as a curing agent through CuAAC reaction. Figure 5 is the non-isothermal differential scanning calorimetric (DSC) curves of polytriazole polybutadiene elastomers (PT_ri_PB) prepared via CuAAC crosslinking reaction between PTPB and 1,6-diazide hexane. It is clear that there only exists a step peak at approximate −75 °C for all PT_ri_PB elastomer S1–S6, which are corresponding to the glass transition temperature of polybutadiene chains [21]. The glass transition temperatures are almost independent of elastomer R values.

### 3.5. Mechanical Properties

**Figure 6:**
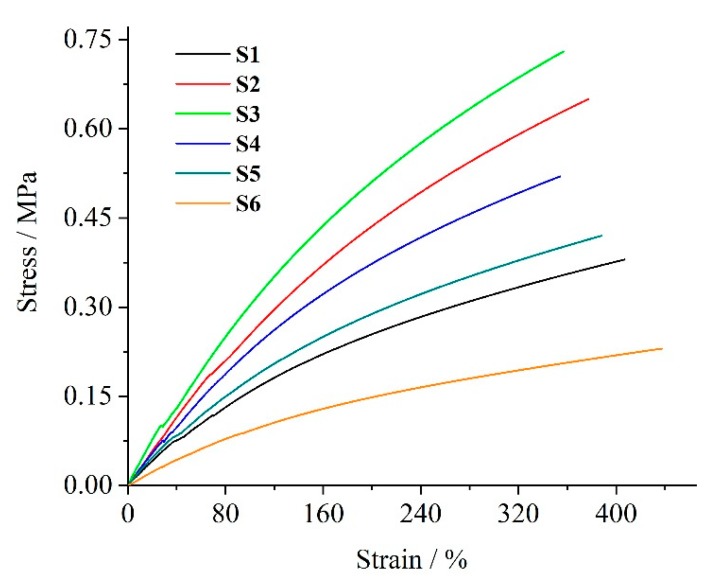
Stress–strain curves of elastomers.

At room temperature, well above their glass transition temperature, the mechanical properties of PT_ri_PB elastomer S1–S6 were measured and are listed in Table 3. It can be observed that the stress at break (*σ*_b_) first increases from 0.39 ± 0.02 MPa to 0.70 ± 0.05 MPa and then decreases to 0.24 ± 0.03 MPa, with increasing *R*, respectively. In contrast, the strain at break (*ε*_b_) first decreases from 396 ± 18 to 329 ± 24% and then increases to 460 ± 18%. The mechanical property maximum and minimum values simultaneously appear at *R* = 1.0 (the stoichiometric ratio). Figure 6 shows the typical stress–strain curves of elastomers. It can be observed that the moduli of elastomers first increase and then decrease as a function of *R* values. Moreover, elastomer S3 with *R* = 1.0 has maximum moduli. The dependence of the mechanical properties of PTPB elastomers on *R* presents parabolic.

**Figure 7:**
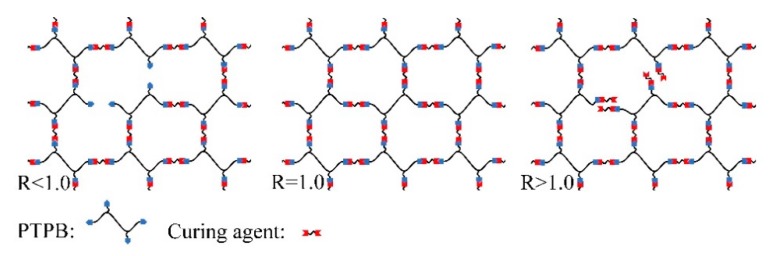
PT_ri_PB elastomer network structures.

Considering that CuAAC reaction involves no side-reactions, PT_ri_PB elastomer network structures can be conceived to be those schematically shown in Figure 7, to further elucidate the mechanical properties [7]. When *R* < 1.0, the terminal propargyl content of PTPB is in excess and propargyl groups cannot be completely reacted with azide groups of curing agent 1,6-diazide hexane, causing the formation of many dangling strands in the elastomer network. Consequently, the elastomers give higher apparent effective strand average molecular weight (*M*_s_) and lower apparent effective strand densities (*N*_0_). At the stoichiometric ratio, the terminal propargyl content is equivalent to that of the azide groups, and terminal propargyl groups are nearly reacted to completion with azide groups of the curing agent. The resultant network structure approaches that of an integrated lattice, with few propargyl and azido groups in the elastomer (see Figure 7; *R* = 1.0); additionally, *M*_s_ is minimized and *N*_0_ is maximized. When *R* > 1.0, the excess azido groups in the curing agent molecules cannot also be reacted with and are still appended as branches, forming larger meshes. Resultantly, *M*_s_ again increases and *N*_0_ again decreases. 

According to Flory elasticity theory, the stress and modulus of crosslinked elastomer are proportional to the density of network chains *N*_0_. The greater the density of network chains, the higher the stress and modulus of crosslinked elastomer, and vice versa [22,23]. Consequently, PT_ri_PB exhibits maximum stress and modulus at the stoichiometric ratio because of its maximum network chain density, whereas its minimum strand molecular weight causes the lowest strain at break. Therefore, the dependence of the mechanical properties of PTPB elastomers on *R* presents as parabolic (see Table 3). 

### 3.6. Thermal Stability

**Figure 8:**
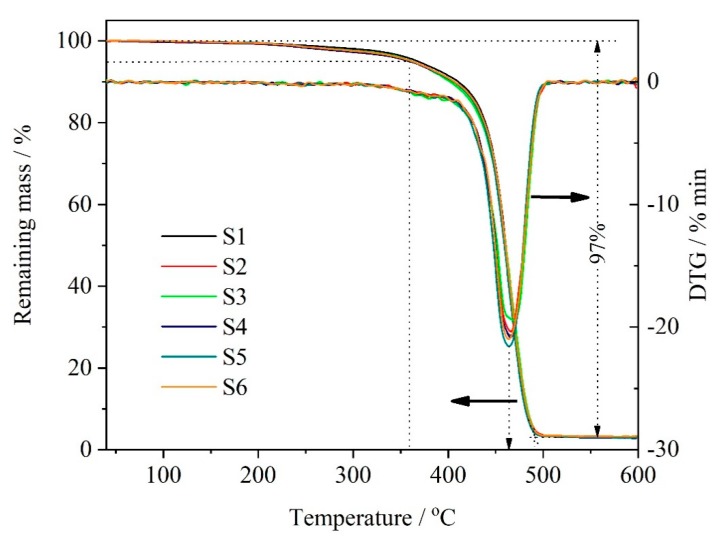
TG and DTG curves for elastomers.

A thermal gravimetric analysis was performed in order to assess the thermal stability of PT_ri_PB elastomers, and their thermal gravity analysis (TG) and derivative thermogravimetric analysis (DTG) curves are shown in Figure 8. It is clear that all TG and DTG curves are nearly overlapped, respectively. The thermal weight loss initiates at 369 °C (T_5%_) and finishes at 485 °C, and the accompanying weight loss is approximately 97%. All DTG curves present the only peaks, indicating that the elastomers have a one-step decomposition characteristic. The weight loss rates reach a maximum at about 463 °C, which is characteristic of polybutadiene thermal decomposition [24]. 

The thermal cleavage temperature of the triazole groups that formed from azide and propargyl cycloaddition reaction is high up to nearly 570 °C [25,26], so the thermal weight loss process of PT_ri_PB elastomer should only be attributed to the thermal degradation of polybutadiene strands rather than the polytriazole crosslinks. The thermal stability of the PT_ri_PB elastomers depends on the polybutadiene strands, but not the polytriazole crosslinks. Additionally, because the fission temperature of urethane bonds usually lies in the range of 300–400 °C [27], the thermal stability of polytriazole crosslinked polybutadiene elastomers should also be advantageous over that of traditional polyurethane ones. 

## 4. Conclusions

Propargyl terminal polybutadiene (PTPB) was successfully synthesized through terminal hydroxyl polybutadiene (HTPB) end-capping modification. PTPB has low viscosity because of no hydrogen bonding interaction among the polymeric chains, which is beneficial to the processing performance of the elastomer preparation.

Using 1,6-diazide hexane as a curing agent, we prepared the PT_ri_PB elastomers with various functional molar ratios (*R*) via CuAAC reaction. The glass transition temperatures of PT_ri_PB elastomers are approximately −75 °C, and the *R* values hardly affect these values. The mechanical properties of the elastomers parabolically depend on the *R* values. At the stoichiometric ratio, the stress and modulus give maximum, while the strain gives minimum, simultaneously. 

The PT_ri_PB elastomer is characteristic of a one-step thermal decomposition process, and its thermal stability depends on the polybutadiene strands, instead of the crosslinking triazole groups. These findings will help researchers discover the more extensive application of CuAAC in the field of elastomer.

## Figures and Tables

**Table 1:** PT_ri_PB elastomer components in gram.

Sample	*R*	Catalyst	Prepolymer PTPB	1,6-diazide hexane
S1	0.8	0.005	10	0.44
S2	0.9	0.005	10	0.50
S3	1.0	0.005	10	0.55
S4	1.1	0.005	10	0.61
S5	1.2	0.005	10	0.66
S6	1.3	0.005	10	0.72

**Table 2:** Parameters of prepolymer HTPB and PTPB.

Sample	Mn	Mw	Mz	Polydispersity
HTPB	6588	13,979	28,126	2.12
PTPB	6678	14,082	29,139	2.11

**Table 3:** Mechanical properties of PTPB elastomers.

Sample	S1	S2	S3	S4	S5	S6
Stress, *σ*_b_/MPa						
1	0.37	0.55	0.63	0.50	0.42	0.30
2	0.38	0.56	0.73	0.58	0.39	0.23
3	0.43	0.65	0.67	0.52	0.39	0.23
4	0.38	0.68	0.78	0.60	0.30	0.20
Average	0.39 ± 0.02	0.61 ± 0.06	0.70 ± 0.05	0.55 ± 0.04	0.38 ± 0.04	0.24 ± 0.03
Strain, *ε*_b_/%						
1	370	370	328	342	388	472
2	387	344	357	321	356	484
3	420	377	281	354	400	438
4	407	329	349	362	360	445
Average	396 ± 18	355 ± 19	329 ± 24	345 ± 13	376 ± 18	460 ± 18
